# Cases of Injuries of the Head, Treated at the Middlesex Hospital

**Published:** 1827-02

**Authors:** 


### INJURIES OF THE HEAD.

### *Cases of Injuries of the Head, treated at the* Middlesex Hospital.

(Continued from page 24.)

In our last Number, we gave some cases of injury of the scalp. These cases show how much the contents of the skull are influenced by the condition of its external coverings, and illustrate the mutual connexion between the scalp, the bone, the brain, and its membranes.

We then proceeded to detail some cases of concussion, and two cases of inflammation of the brain, the remote effect of this injury, were related, with a view to show the masked form which inflammation of the brain frequently assumes after this accident. A fatal case of concussion was also given, and the morbid appearances presented on dissection were described.

We now subjoin another case of concussion, in order to show its several stages, and the utility of the division laid down by Mr. Abernethy.

Case VI. *Concussion of the Brain.* Treated by Mr. Joberns.

Charles Cox, ætatis eleven, admitted December 6th, 1826. This boy had fallen thirty feet: he was taken up senseless, and was conveyed to a surgeon, who bled him. When brought here, he still remained in the same state of insensibility, although an hour had elapsed since the occurrence of the accident. He was cold, pale, and pulseless; the pupils were dilated; and his breathing was tranquil. There was a wound of the scalp near the vertex and over the right parietal bone, which was bared of its pericranium. The denuded portion of bone might be somewhat larger than a shilling. In less than an hour he began to show signs of returning sense. He cried violently when his head was touched; but, when left alone, he sunk into his former state of stupor. He lay curled up; his knees and thighs were drawn towards the abdomen, and his head was bent forwards, and hid under the bedclothes. He complained of his right leg, which being examined, the tibia was found to be fractured. His pulse now became more perceptible, and the heat of the surface was returning.

The head was shaved. The dirt was cleared from the wound; the divided parts were brought together by adhesive straps, and the head was covered with an evaporating lotion. He took Calomel gr. iij. with Jalap gr. xij.

7th.—Much in the same state; moaning a good deal, cross and irritable. His pulse had risen somewhat, but was still very weak. As the purgative had not yet operated, he was ordered repeated doses of the house medicine until the bowels were acted upon. By the evening the bowels had been freely opened, but he had passed both fæces and urine in bed. The following mixture was directed to be taken three times a-day:

H. Potassæ Tart. ℥ jss.; Vin. Antim. T. ʒ ss.

8th.—Has been noisy during the night; talks incoherently; there is a dropping of the eyelids, but he says that he has no pain. Pulse about seventy-five, and weak. The wound has partially adhered.

Hirudines xiv. to be applied to the temples.—Calomel gr. iij. Antim. Tart. gr. $\frac{1}{4}$, to be taken at bed-time.

9th.—He does not sleep, but is crying out continually. He is extremely obstinate, and says that he "will make a noise." He still passes his fæces and urine in the bed, and seems inclined to do any thing but what he is bid. The pulse, although weak, communicates to the finger a peculiar vibratory feel.

Hirudines xij. to be applied to the temples, and to take a dose of Calomel and Jalap.

10th.—The face has become flushed; the skin is hot and dry; and the tongue, for the first time, differs from its healthy condition: it is now covered with a yellowish-white fur, its edge is red. The pulse has the same thrilling feel as yesterday; it is more accelerated, (about eighty-five,) and has acquired some degree of strength.

He was bled to four ounces, and twelve leeches were applied to the temples.

12th.—Continues very noisy, and does not sleep. The pulse was rendered soft by the last bleeding, but to-day has again assumed its former vibratory character.

Six ounces of blood were taken from the arm.

14th.—He improves; slept better last night, and is more rational to-day; cries out less, but is still peevish and irritable. There has been no intolerance of light from the first, and he has never once complained of pain in the head, unless the wound was touched. Pulse eighty, and less vibratory; tongue moist, and covered with a white fur. The adhesions of the wound had all given way, and the bone was found to be rough and bare to the extent already mentioned.

The wound was covered with a poultice, and twelve leeches applied to the temples.

16th.—There is little alteration in the symptoms since our last report. The tongue has begun to clean towards its edges; the pulse has not lost its thrilling character.

Bled to seven ounces.

19th.—He is less irritable, and more under control; he sleeps better, is not so noisy, and is more civil in his answers to questions. Tongue clean; pulse full and regular.

His amendment was now progressive; the wound suppurated kindly, but the bone continued bare. He had no recollection of the accident.

January 5th.—The scalp has united to the bone, and the parts of the wound are all consolidated. He remains in the hospital for the fractured tibia, which has also proceeded very favourably.

In our last Number we gave a case of fatal concussion, in which stertorous breathing was one of the most prominent symptoms. This has been considered by many as a diagnostic symptom of compression. In the case alluded to, however, there was no compression, (at least in its first stage,) yet the stertor was well marked. The following case will serve to show, on the other hand, that there may be considerable extravasation of blood on the brain, and still the breathing may be tranquil.

*Case of Extravasation of Blood between the Skull and the Dura Mater.*

Richard Smith, ætatis ten, was brought to the Middlesex Hospital, August 24th, 1826, labouring under all the usual symptoms of compression of the brain. It had been caused, as we were informed, by a heavy piece of timber, which, sliding from its resting place, had crushed the boy to the ground. He remained insensible for a few minutes after the accident, and he vomited several times before he was brought to the hospital.

He was pale, cold, and motionless; his pulse was small and feeble; and he was constantly vomiting. On examining the head, a contusion of the scalp was found at about the middle of the parietal bone: there was no wound. The fingers were pressed in upon the tumid scalp, and the bone was carefully examined, but no irregularity in the skull could be detected. From the symptoms present, it was evident that the brain had received a most serious injury. The dangerous condition of the child was represented to the parents, but all our intreaties could not prevail upon them to allow the child to remain in the hospital, and he was therefore visited at home by the house surgeon.

The head was shaved, and the following draught was ordered to be taken every eight hours:—Magnes. Sulph. ʒj.; Vin. Antim. Tart. m. xx.; Aquæ Puræ ℥j.—The head was covered with an evaporating lotion.—Low diet.

25th.—His bowels had been well opened, but he still lay in the same comatose state as yesterday,—with this difference, however, in the general symptoms, that the skin, which was before pale and chilly, had now become hot and dry; and that the pallid and death-like countenance had given place to the flush and suffusion of general vascular excitement. The pulse was no longer feeble and intermitting, it had become quick and strong, but it was not more frequent than natural. *The breathing was perfectly easy.* The irides acted irregularly, alternately contracting and dilating. If spoken to in a loud tone, and shaken, he could be roused so as to answer questions, but again immediately relapsed into the same state of lethargy. His thighs were drawn up towards the abdomen, and his legs were bent upon his thighs: thus he lay as in a deep sleep. He asked for drink, &c. or for the chamber-pot, if he at any time needed them. He would sometimes roll about in his bed, and moan; and he frequently raised his hands to his head. He was impatient if any attempts were made to open the eyelids, and drew his head away when the injured part of the scalp was touched.

The excited state of the vascular system seemed to call for venesection, and ten ounces of blood were taken from the arm before any manifest alteration in the pulse was produced. After the bleeding, his senses seemed to be partially restored: he opened his eyes, sat up in bed, and spoke to those around him. This favourable change was not of long continuance, he soon again became more insensible. As the vomiting still continued, the antimony was omitted, and the sulphate of magnesia was given in smaller doses.

26th.—He was much more sensible than before the bleeding: he answered questions, and did not complain of pain. He still lay moaning, and regardless of surrounding objects, but he was not so deeply comatose as yesterday; pulse weak, and rather frequent. At four o'clock in the afternoon he became convulsed, and died.

*Dissection.*—The scalp was turned back, and a quantity of blood was found in the cellular texture under it, where it covers the anterior part of the left parietal bone. Here the parietal bone was fractured, midway betwixt its anterior angles and about an inch posterior to the coronal suture; a small portion of the bone was slightly depressed. From this part a fissure might be traced, which ran in a direction backwards, traversing the posterior part of the parietal bone, and terminating near the base of the skull.

On removing the calvarium, about two ounces of dark coagulated blood were found on the dura mater, under the fractured and depressed portion of the skull; and now it was discovered that two of the main branches of the middle meningeal artery had been torn, yet the dura mater was not perforated. That part of the left hemisphere of the brain upon which the coagulum had pressed was flattened, and appeared to be rather softer in its texture than the surrounding parts. There was a dark spot on the surface of the brain, which corresponded to the depressed portion of bone. There was nothing remarkable in the vascular system of the brain, no injection of the minute vessels, no signs of inflammation.

The above case was accompanied by a train of symptoms of very common occurrence. The patient lies with his legs drawn up towards the abdomen, and with his head and neck bent forwards; his trunk and extremities are thus, as it were, rolled and packed up in the smallest possible compass. He lies as if in a profound sleep, and is averse to being disturbed. You can rouse him so as to answer your questions, but his answers are generally in monosyllables. He gets out of bed to pass his evacuations; and not unfrequently he makes water against the wall, or passes his stool on the floor. He asks for drink, or whatever he may want. *There is no stertor.* The eyelids are closed, and he resists your attempts to open them. The irides are irregular in their action, being at one moment contracted, and at another dilated. Whether these symptoms be those of compression or of concussion, or of either, has not yet been ascertained. Experience has failed to point out the symptoms which distinguish these two affections from one another. Mr. John Bell, with his accustomed felicity of description, has given the above peculiar train of symptoms as indicative of compression; whilst, on the other hand, Sir Astley Cooper has traced them to concussion. In short, no correct diagnosis between compression and concussion has yet been made; so that in another place Mr. J. Bell has it, that "concussion is, like compression, an affection of the whole brain, and, in so far as we know it by symptoms, it is entirely the same." Mr. Abernethy also tells us, that, "in cases where there is no interval of sense after the accident, we are at a loss to determine whether the senseless state be the effect of compression or concussion."

Thus, if any inference can be drawn from the cases here related, and from numerous others of a similar kind, we should be induced to say that these symptoms arise from the general injury done to the brain, whether that injury be caused by compression, concussion, or laceration of the brain; and that it is only by the state of the pulse, the duration of the symptoms, and by a minute attention to the general circumstances of the case, that we can hope to distinguish between compression and concussion of the brain.

Case VIII. *Injury of the Head, attended by Symptoms similar to those of the preceding, and which seemed to indicate Compression of the Brain.* Treated by Mr. Joberns.

Patrick Cawdy, a strong Irish labourer, was brought to the hospital July 12th, 1826. He had fallen from a scaffolding, a height of thirty-five feet. He was quite insensible; the pulse was scarcely to be felt; the pupils were widely dilated; but respiration was performed with freedom. He rolled about in the bed, groaned frequently, and often put his hands to his head. If you attempted to open his eyelids, or otherwise disturbed him, he became restless and impatient: he tried to keep his eyelids closed, and drew his hand away when you took it to feel his pulse. He lay with his legs and thighs drawn up towards the abdomen, his head depressed towards his chest.* The scalp appeared to be uninjured, and the only external mark of injury to the head was a bleeding from the left ear, the hemorrhage from which was unusually great.

The head was shaved, and he took a purge of Calomel and Jalap.

His pulse continued weak and slow all day, it did not exceed forty in the minute. If you shook him and attempted to rouse him, he seemed to be annoyed, muttered indistinctly, and drew himself away, and was not satisfied until allowed to resume his former position. He then fell into the same profound sleep as before.

13th.—He continued much in the same state. The pulse was still slow and weak; the pupils were less dilated. As the calomel and jalap had not yet operated, it was repeated, and a common purgative enema was administered. Twelve leeches were applied to the temples; and, as the heat of the scalp had increased, the head was covered with an evaporating lotion.—In the evening, the pulse was at fifty, and had acquired a little more strength. His bowels had been opened during the day, and he had got out of bed to pass his urine, &c. He opened his eyes for the first time, and when roused would speak; but he again instantly fell into his previous state of lethargy.

A blister was applied to the nape of the neck.

14th.—He was somewhat more sensible. You could occasionally, by dint of perseverance, extract answers to your questions, but the answers were generally given in monosyllables; he rolled about in his bed, and talked incoherently. The pulse was somewhat stronger than on the preceding night; the pupils were more contracted.

He took another dose of Calomel and Jalap, and had an aloetic enema.

In the evening, as the pulse had become quicker and less compressible, he was bled to eight ounces, which had the effect of lowering the pulse considerably. During this operation he yawned frequently, opened his eyes as if awoke from a sound sleep, and stared idiotically on those around him.

15th.—The sensorial functions were performed in the same imperfect manner as on the preceding day. He complained of a pain in his head, and his pulse was more frequent; the bowels had not been relieved.

The aloetic glyster was repeated, and he took doses of the house medicine until free evacuations were procured. Ten leeches were applied behind the ears; and he was to take Calomel gr. iij. Antimonial Powder gr. j. three times a-day.

17th.—He had certainly improved: he had become more rational, and was less lethargic. Still he did not seem inclined to

* When the injury to the brain is not so great as to prove quickly fatal, the patient, as far as our experience goes, almost always lies in the position above described.

answer questions; he was sulky, and grumbled when he was disturbed.

19th.—To-day, for the first time, we succeeded in getting him to put his tongue out: it was foul and furred, which condition was probably in part owing to the mercury that he had taken. He still continued to moan and toss about in his bed, and was constantly putting his hands to his head. His bowels were preserved in a lax state by the house medicine and aloetic glysters. He had taken little more than milk and water-gruel since his admission.

22d.—His mouth appeared to be very sore, although he denied that it was so. The calomel and antimony were discontinued. To-day he sat up for the first time. He said that he felt a pain in his forehead. He was sullen, and spoke only when spoken to; he recognised some of his friends.

Fourteen leeches were applied to the temples.

This man continued much in the same state for some time. He constantly complained of his head; he said that he was dizzy, and felt a weakness in the fore part of his head. The treatment consisted in a perseverance in the antiphlogistic means already enumerated,—namely, in local bleeding, blistering, purgative and antimonial medicines.

August 5th.—He was well enough to walk in the garden with the assistance of another man, but, if he attempted to walk alone, he staggered like one drunk.

He was discharged the hospital August 15th. At that time he could walk without assistance; he was quite deaf in the left ear; his intellects were much impaired; his countenance was a complete blank; in short, he was little better than an idiot.

We have since seen him, and found that the sensorial functions were beginning to be restored. 141

